# A Super-Resolution and 3D Reconstruction Method Based on OmDF Endoscopic Images

**DOI:** 10.3390/s24154890

**Published:** 2024-07-27

**Authors:** Fujia Sun, Wenxuan Song

**Affiliations:** School of Mechanical Engineering, University of Shanghai for Science and Technology, Shanghai 200093, China

**Keywords:** image super-resolution, endoscopic image processing, 3D reconstruction, structure from motion, multi-view stereo

## Abstract

In the field of endoscopic imaging, challenges such as low resolution, complex textures, and blurred edges often degrade the quality of 3D reconstructed models. To address these issues, this study introduces an innovative endoscopic image super-resolution and 3D reconstruction technique named Omni-Directional Focus and Scale Resolution (OmDF-SR). This method integrates an Omnidirectional Self-Attention (OSA) mechanism, an Omnidirectional Scale Aggregation Group (OSAG), a Dual-stream Adaptive Focus Mechanism (DAFM), and a Dynamic Edge Adjustment Framework (DEAF) to enhance the accuracy and efficiency of super-resolution processing. Additionally, it employs Structure from Motion (SfM) and Multi-View Stereo (MVS) technologies to achieve high-precision medical 3D models. Experimental results indicate significant improvements in image processing with a PSNR of 38.2902 dB and an SSIM of 0.9746 at a magnification factor of ×2, and a PSNR of 32.1723 dB and an SSIM of 0.9489 at ×4. Furthermore, the method excels in reconstructing detailed 3D models, enhancing point cloud density, mesh quality, and texture mapping richness, thus providing substantial support for clinical diagnosis and surgical planning.

## 1. Introduction

In modern medical imaging, high-quality image reconstruction is crucial for ensuring the accuracy and efficiency of clinical diagnoses [1]. In minimally invasive surgery, endoscopic examinations have become indispensable tools, and the clarity of endoscopic images directly determines whether doctors can accurately identify pathological tissues and thus formulate effective treatment plans [2]. However, due to the physical and technical limitations of endoscopic equipment, these images often suffer from low resolution and noise interference. These issues severely affect image quality, thereby limiting their effectiveness in clinical applications.

Although traditional image super-resolution techniques have made significant progress in many fields, research on endoscopic image processing remains relatively scarce [3]. These techniques often fail to fully consider the highly textured characteristics of endoscopic images and the need to capture details and control noise when processing these images [4,5]. Furthermore, existing studies on endoscopic 3D reconstruction often rely on the quality of the original images and do not systematically integrate super-resolution processing with 3D modeling technology, limiting the accuracy and practicality of reconstructed models [6].

In recent years, despite the advancements made by traditional super-resolution techniques in endoscopic image processing, such as the SRCNN method proposed by Dong et al. [7], these methods typically struggle to handle the complex textures and high levels of noise present in endoscopic images [8]. To address this challenge, emerging deep learning methods, particularly convolutional neural network-based super-resolution techniques, have been developed. For instance, Kim et al. [9] proposed the DRCN method, which learns the residuals between low-resolution and high-resolution images (i.e., the detail differences), rather than directly learning the output images themselves, thus improving training efficiency and output image quality. Liang et al. [10] introduced SwinIR, which leverages a hierarchical Transformer structure to handle features of varying scales in images, enhancing the precision in detail recovery and overall image quality. Lim et al. [11] removed batch normalization layers from traditional residual networks to improve super-resolution performance, showing excellent results in processing images with rich details and textures. Zhang et al. [12] employed a channel attention mechanism to enhance the network’s capability in expressing features, particularly for high-frequency details, significantly boosting the model’s performance through the use of attention mechanisms. Wang et al. [13] introduced the Omni Self-Attention (OSA) and multi-scale interaction scheme to address the limitations of traditional self-attention by promoting comprehensive interaction in both spatial and channel dimensions, significantly enhancing performance while maintaining minimal computational budget and achieving state-of-the-art results in lightweight super-resolution benchmarks.

In the realm of 3D reconstruction of endoscopic images, Schonberger et al. [14] proposed a new SfM (Structure from Motion) technique, suggesting improvements in the robustness, accuracy, completeness, and scalability of SfM systems. Gao et al. [15] employed a fusion method of SIFT and SURF feature extraction results, enhancing the robustness of feature matching by increasing the number of feature points and applying the RANSAC algorithm. Liu et al. [16] improved the spatial distribution and connectivity management within subgroups by developing a multifactorial joint scene partitioning metric and a pre-allocation balanced image expansion algorithm, addressing issues of loose spatial distributions within subgroups and enhancing inter-subgroup connectivity. Liu et al. [17] combined multi-view stereo techniques, semantic plane detection branches, and plane MVS branches, increasing the accuracy of plane detection and 3D geometric measurements from multiple viewpoints. Jia et al. [18] developed a Transformer-based coarse-to-fine granularity multi-view stereo matching network specifically for 3D reconstruction of low-resolution images, enhancing the accuracy and completeness of 3D point clouds by aggregating global contextual information through Transformer modules. At the same time, Chen et al. [19] applied Gaussian scattering techniques to reconstruct deformable soft tissue structures from endoscopic videos, enhancing the accuracy of 3D reconstructions and reducing misjudgments of soft tissue parts, thereby improving the credibility and practicality of the reconstructed results. Batlle et al. [20] utilized the characteristics of artificial lighting environments within the human body to provide a simple calibration process suitable for real medical environments, achieving 3D information acquisition from a single perspective under controlled lighting conditions by considering light sources associated with camera movement. Ahmad et al. [21] employed shape-from-shading algorithms to reconstruct 3D shapes from single images, using light and shadow variations in endoscopic images to infer the 3D structure of the scene. However, these studies overlook the impact of the intrinsic quality of endoscopic images on the reconstruction outcomes.

In summary, current 3D reconstruction technologies mainly focus on rigid objects, whose physical properties allow them to maintain structural integrity during observation [22]. This emphasis on rigid objects [15] presents numerous challenges when applying existing 3D reconstruction methods to endoscopic images, which often involve complex flexible tissues. These tissues may deform during medical procedures, rendering traditional rigid reconstruction algorithms unsuitable [23]. Additionally, the unique aspects of endoscopic imaging, such as limited field of view, uneven lighting conditions, and inherent blurriness of the images, further complicate the 3D reconstruction process, which has been largely overlooked in existing research [24].

In order to respond to these challenges, this paper has carried out corresponding optimization work on endoscopic images. The specific contributions are described as follows:

(1) The Omni Self-Attention (OSA) mechanism, Omni-Scale Aggregation Group (OSAG), Dual-stream Adaptive Focus Mechanism (DAFM), and Dynamic Edge Adjustment Framework (DEAF) are employed to enhance the accuracy and efficiency of super-resolution processing. These technologies significantly improve the model’s effective receptive field and contextual aggregation capabilities, especially when dealing with endoscopic images that feature complex textures and fine edges.
(2) Combining Structure from Motion (SfM) and Multi-View Stereo (MVS) technologies, high-precision medical 3D models are obtained. These techniques accurately reconstruct detailed 3D models, significantly enhancing point cloud density, mesh quality, and the richness of texture mapping.

## 2. Materials and Methods

### 2.1. Image Super-Resolution Algorithm

To overcome the limitations of traditional algorithms in processing highly textured medical images [25], the OmDF-SR algorithm has been optimized to meet the unique requirements of endoscopic image super-resolution. These limitations mainly include the restricted receptive field of models when processing images of complex tissue structures and their inadequacies in multi-scale feature aggregation. By introducing the Omni Self-Attention (OSA) mechanism, Omni-Scale Aggregation Group (OSAG), Dual-stream Adaptive Focus Mechanism (DAFM), and Dynamic Edge Adjustment Framework (DEAF), the algorithm significantly enhances the effective receptive field and contextual aggregation capabilities of the model.

#### 2.1.1. OSA

**Figure 1:**
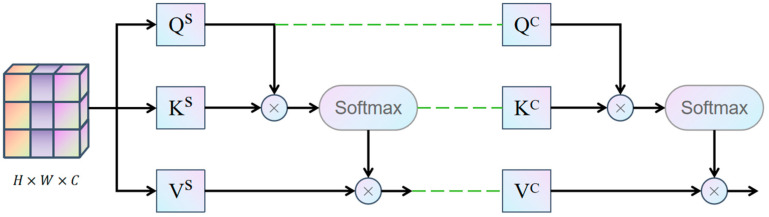
The structure of Omni Self-Attention (OSA).

The OSA mechanism is the core of the OmDF-SR framework. It extends the traditional self-attention mechanism to consider both spatial and channel information during feature aggregation. This comprehensive information processing method is crucial for capturing details in endoscopic images, especially when dealing with images that have complex textures and fine edges. By effectively integrating spatial and channel attention, OSA significantly enhances the model’s ability to capture these details, as illustrated in Figure 1.

OSA first maps the input feature map into Query (Q), Key (K), and Value (V) matrices through a series of linear transformations:

(1) $$Q=XW^{Q}.K=XW^{K},V=XW^{V}$$

where $W^{Q},W^{K},\;and\;W^{V}$ are the learned weight matrices.

This process takes into account not only the spatial relationships between pixels but also the dependencies between different channels. By computing the dot product of Q and K, and then applying the Softmax function, OSA generates an attention map that guides how information in the V matrix is aggregated.

(2) $$A_{s}=SoftMax(\frac{QK^{T}}{\sqrt{d_{k}}})$$

Here, $d_{k}$ is the dimension of the key vectors, used to scale the dot product results to prevent excessively large values from affecting the gradients of the Softmax function.

Additionally, after computing spatial attention, OSA transposes the feature matrix to perform channel attention calculations, further enhancing the model’s ability to capture global information. Similar to spatial self-attention, channel attention also computes the dot product, but it is performed along the channel dimension:

(3) $$A_{c}=SoftMax(\frac{K^{T}Q}{\sqrt{d_{k}}})$$

Here, $d_{k}$ is the dimension of the query vectors. The outputs of spatial and channel attention are combined through weighted value matrices and processed by a nonlinear activation function:

(4) $$Y=\sigma (A_{s}V+A_{c}V)$$

#### 2.1.2. OSAG

**Figure 2:**
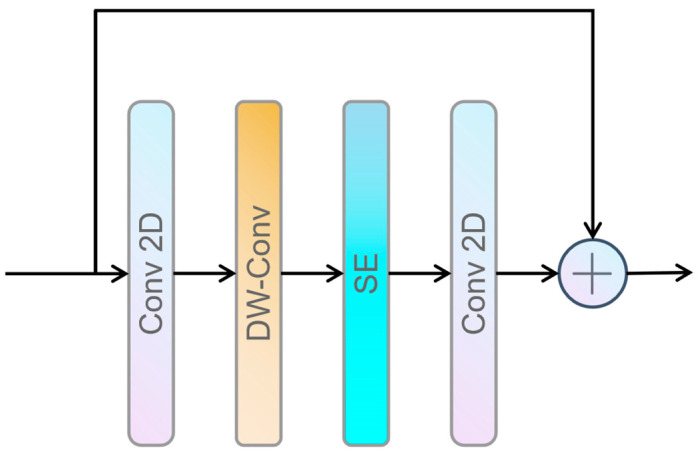
The structure of Local Convolution Block (LCB).

To overcome the limitations of traditional models in handling features of different scales, OmDF-SR introduces OSAG. OSAG achieves multi-scale feature processing through a three-level aggregation strategy: local, mid, and global. This effectively expands the model’s effective receptive field. The local level uses an enhanced inverted bottleneck structure to process local patterns, which helps to handle local patterns at low cost, as shown in Figure 2.

In this figure, Conv 2D represents a 2D convolutional layer, DW-Conv stands for the depthwise separable convolutional layer, and SE denotes the squeeze-and-excitation module.

The mid-level performs attention operations within a set of non-overlapping blocks, focusing on medium-scale pattern recognition. Specifically, the non-overlapping blocks are sized to P×P, and the dimensions are rearranged to facilitate medium-scale interactions:

(5) $$\left(H,W,C)\to (\frac{H}{P},\frac{W}{P},C)\to (\frac{HW}{P^{2}},P^{2},C\right)$$

The global level sparsely samples the entire feature map to achieve global interactions, using dilation to process global information. The input features are divided into a G × G grid, with each grid point dimensioned adaptively to $\frac{H}{G}\times \frac{W}{G}$:

(6) $$\left(H,W,C)\to (G\times \frac{H}{G},G\times \frac{W}{G},C)\to G^{2},\frac{HW}{G^{2}},C\right)$$

This hierarchical feature aggregation strategy can enhance the quality of image reconstruction and optimize the computational efficiency of the model. With this design, OmDF-SR is able to achieve performance comparable to larger models while maintaining lower computational complexity.

#### 2.1.3. DAFM

**Figure 3:**
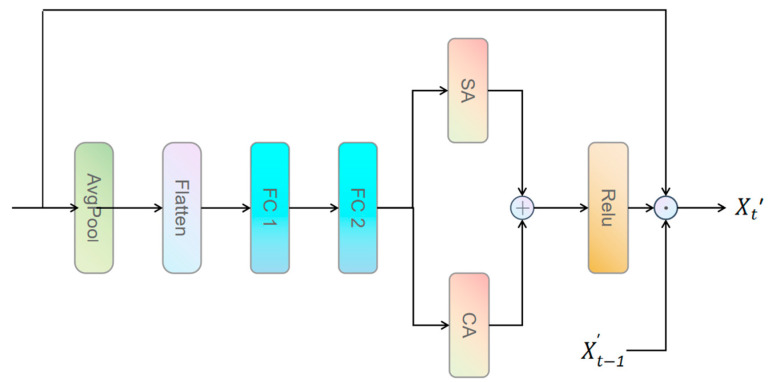
The structure of the Dual-stream Adaptive Focus Mechanism (DAFM).

To further enhance the algorithm’s ability to process complex textures and subtle edges in endoscopic images, this study proposes the Dual-Stream Adaptive Focus Mechanism (DAFM). This mechanism aims to enhance the OmDF-SR algorithm’s capability to handle intricate image textures and edges. DAFM combines spatial attention and channel attention, adaptively adjusting their weight distribution to achieve optimal feature aggregation, as illustrated in Figure 3.

The DAFM predicts the fusion weights of spatial and channel attention through a fully connected network, optimizing the selective enhancement of features. It applies global average pooling to the input features to reduce the number of parameters and improve computational efficiency and then uses the Softmax function to ensure the normalization of the output weights. The fused attention map is obtained by a weighted combining of spatial and channel attention, processed through a nonlinear activation function to enhance the fused representation. This enhanced representation is then applied to the input feature map. Through an adaptive feedback mechanism, the attention distribution is dynamically adjusted, improving the model’s adaptability to changes in continuous image sequences:

(7) $$W=Softmax(W_{f}^{2}\cdot ReLU(W_{f}^{1}\cdot Flatten(AvgPool(X))+b_{f}^{1})+b_{f}^{2})$$

(8) $${X^{\prime }}_{t}=\sigma (X⊙ReLU(ReLU(W_{0}\cdot A_{s})+ReLU(W_{1}\cdot A_{c}))+\beta \cdot {X^{\prime }}_{t-1})$$

Here, AvgPool represents global average pooling, Flatten denotes the conversion of multi-dimensional inputs into a one-dimensional array, $W_{f}^{1},W_{f}^{2},b_{f}^{1},\;and\;b_{f}^{2}$ represent the weights and biases of the two layers of the network, respectively, and β is the feedback coefficient.

#### 2.1.4. DEAF

To address the issue of blurriness and noise in the edge regions of endoscopic images, we integrated a Dynamic Edge Adjustment Framework (DEAF) into the network. The DEAF enhances the visual clarity and detail perception of the images by precisely adjusting the feature responses in the edge regions, making the edges sharper and easier to recognize, which is crucial for endoscopic images.

OmDF-SR performs edge detection using adjusted convolution kernels, emphasizing high-frequency details by increasing the core weights to highlight the image edges. The detected edge map is calculated through the following convolution operation:

(9) $$E=W_{e}*X+b_{e}$$

Here, $W_{e}$ represents the weights of the edge detection convolution kernel and $b_{e}$ denotes the bias.

The detected edge map is combined with the original input feature map to enhance edge contrast and improve image quality:

(10) $$X^{\prime }=X+E$$

#### 2.1.5. Reconstruction

The high-resolution image $I_{HR}$ is reconstructed by aggregating shallow and deep features:

(11) $$I_{HR}=H_{REC}(X_{0}+X_{DF})$$

Here, $H_{Rec}\left(\cdot \right)$ represents the reconstruction module, $X_{0}$ is the input low-resolution feature map, and $X_{DF}$ is the deep feature map processed by the OmDF-SR network.

Each level of OSAG includes a Local Convolution Block (LCB), a Meso-OSA Block, and a Global-OSA Block. These modules work together to achieve information integration from local to global, effectively enhancing the model’s ability to capture and restore details. The entire process can be expressed as follows:

(12) $$X_{res}=H_{Global-SPOAB_{i}}(H_{Meso-SPOAB_{i}}(H_{LCN_{i}}(X_{i-1})))$$

(13) $$X_{i}=H_{SEA_{i}}(H_{conv_{i}}(X_{res}+X_{i-1}))$$

Here, $X_{i-1}$ and $X_{i}$ represent the input and output features of the i-th OSAG, respectively.

#### 2.1.6. OmDF-SR Framework

**Figure 4:**
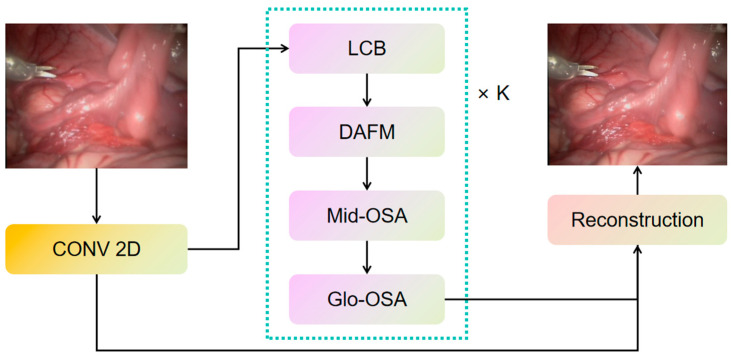
The overall architecture of the proposed OmDF-SR framework.

OmDF-SR optimizes the super-resolution reconstruction process of images by integrating the Omni-Directional Self-Attention mechanism and the Omni-Scale Aggregation strategy, as shown in Figure 4.

#### 2.2. 3D Reconstruction Algorithm

##### 2.2.1. Structure from Motion (SfM)

SfM is used to infer the 3D structure of the scene and the camera motion path from a series of 2D images. This process typically involves the following steps:

Feature Extraction: First, feature points are extracted from each endoscopic image. These feature points should be distinctive corners, edges, or other prominent image features. Common feature extraction algorithms include Scale-Invariant Feature Transform (SIFT), Speeded Up Robust Features (SURF), and Oriented FAST and Rotated BRIEF (ORB):

(14) L(x,y,σ)=G(x,y,σ)∗I(x,y)

Here, L(x,y,σ) is the scale-space function, G(x,y,σ) is the Gaussian kernel, and I(x,y) is the original image.

Feature Matching: Subsequently, the relative motion between consecutive image frames is estimated by finding matching feature points. This matching process typically relies on feature descriptors, which effectively represent the local image information of features. Feature matching is usually implemented using brute-force matching or FLANN (Fast Library for Approximate Nearest Neighbors) matcher, with Euclidean distance as the matching criterion:

(15) $$D(i,j)=\sqrt{{\sum_{1}^{128}\left(d_{i}^{k}-d_{j}^{k}\right)}^{2}}$$

Here, $D\left(i,j\right)$ represents the distance between the i-th and j-th feature point descriptors and $d_{i}^{k}$ and $d_{j}^{k}$ are the k-th elements of the descriptor vectors.

Camera Parameter Estimation: Using the matched feature points, the camera pose and position for each frame relative to the first frame can be estimated by solving a Perspective-n-Point (PnP) problem. This typically involves minimizing the reprojection error, which is the difference between the observed image points and the points projected through the camera model:

(16) $$\min_{R,t}=\sum_{i=1}^{N}||x_{i}-\pi (K,R,t,X_{i})|{|}^{2}$$

Here, $x_{i}$ represents the point in the image, $X_{i}$ is the corresponding 3D point, π is the projection function, K is the intrinsic parameter matrix, and R and t are the rotation and translation parameters, respectively.

Sparse 3D Reconstruction: By combining the estimated camera parameters and matched feature points, a sparse 3D point cloud model is constructed. This model represents a rough 3D structure of the scene captured by the camera.

##### 2.2.2. Multi-View Stereo (MVS)

After obtaining the sparse 3D model, Multi-View Stereo (MVS) is used to generate a more accurate and detailed 3D model. MVS estimates the dense depth and surface information of the scene by analyzing images from multiple viewpoints.

Depth Map Estimation: For each image viewpoint, MVS analyzes neighboring views to determine the depth value of each pixel. This is typically achieved by computing the similarity between pixels, using methods such as Normalized Cross-Correlation (NCC), Windowed Sum of Squared Differences (SSD), or other image similarity metrics:

(17) $$NCC(I_{1},I_{2})=\frac{\sum x,y[I_{1}(x,y)-\mu_{I_{1}}][I_{2}(x,y)-\mu_{I2}]}{\sqrt{{\sum x,y{\left[I_{1}(x,y)-\mu_{I_{1}}\right]}^{2}[I_{2}(x,y)-\mu_{I2}]}^{2}}}$$

Here, $X_{world}$ are the pixel intensities of two neighboring views and $\mu_{I_{1}}\;and\;\mu_{I_{2}}$ are their respective mean values.

Dense 3D Reconstruction: Using the depth maps obtained from each viewpoint, a dense 3D point cloud can be constructed. Each depth value corresponds to a 3D spatial point extending from the camera center to the pixel location according to the depth.

Surface Reconstruction: A continuous surface is extracted from the dense point cloud. This usually involves techniques such as Poisson reconstruction or Delaunay triangulation. This step is crucial for converting the scattered 3D point cloud into a continuous, smooth 3D surface model:

(18) $$Surface=PoissonRecon(X_{dense})$$

**Figure 5:**
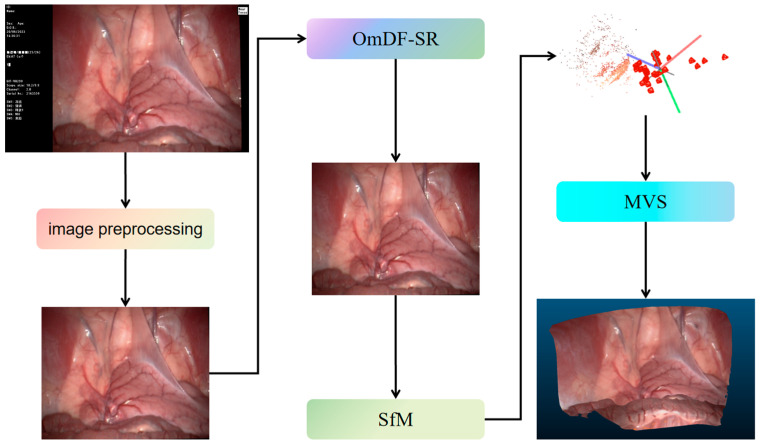
The overall architecture of 3D reconstruction.

The overall framework of the model is illustrated in Figure 5.

## 3. Results

The main objective of this experiment is to verify the impact of using the OmDF-SR algorithm on the efficiency of 3D reconstruction after super-resolution processing of endoscopic images. Endoscopic images are typically limited by the resolution of the equipment and the complex internal lighting conditions, which may result in insufficient image detail. This directly affects subsequent clinical diagnosis and the formulation of treatment plans. We aim to demonstrate the performance of OmDF-SR in enhancing image resolution and improving image quality, particularly in terms of detail recovery and noise suppression. Additionally, the super-resolved images will be used for subsequent 3D reconstruction of stomach models. This paper will evaluate how the super-resolution preprocessing step improves the accuracy and detail representation in 3D reconstruction. Through comparative experiments, we hope to demonstrate the potential contribution of the OmDF-SR algorithm to enhancing the clinical value of endoscopic images and the quality of 3D reconstruction.

### 3.1. Experimental Settings

The dataset used in this study includes partially publicly available datasets [26], as well as a self-collected endoscopic image dataset consisting of 561 highly textured endoscopic images, to ensure diversity and relevance to practical applications. All the original endoscopic images used in this study have a resolution of 640 × 480 pixels, which helps to maintain consistency in super-resolution processing and accuracy in evaluation results. These images reflect the diversity and complexity of real clinical environments, ensuring the practical application value of the study.

The experiments were conducted using the Google Colab platform, with a hardware environment including an NVIDIA Tesla T4 GPU (PNY Technologies, New York, NY, USA), featuring 15 GB of VRAM and CUDA 12.2 support. This hardware configuration provided ample computational resources for complex image processing and model training tasks, ensuring the efficiency and stability of the experiments.

### 3.2. Image Super-Resolution Experiment

**Figure 6:**
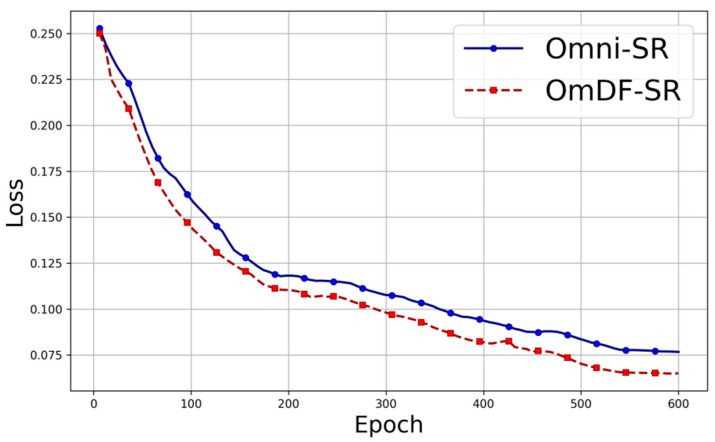
Training loss of different model.

In this study, we conducted a detailed evaluation of the OmDF-SR algorithm, with a particular focus on its performance during the training process and on its effectiveness in the super-resolution processing of endoscopic images. Firstly, we recorded the changes in the loss function during the training process to verify the convergence and stability of the model, and then compared these changes with those of the original algorithm, as shown in Figure 6.

**Figure 7:**
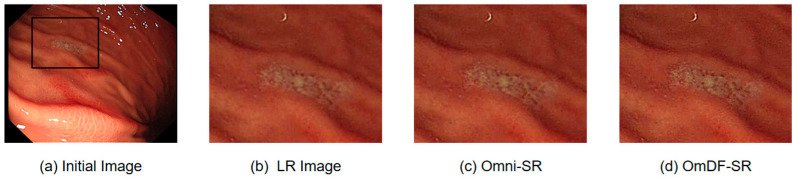
Perceptual results of different models, corresponding HR image, and initial image with enlargement scale factor ×2.

As the number of training epochs increases, the loss value of the model shows a clear downward trend, indicating that the algorithm is gradually learning the key features in the data and effectively optimizing the errors in the super-resolution reconstruction process. The improved algorithm demonstrates a faster loss reduction rate compared to the original algorithm. This suggests that enhancements such as the improved feature aggregation mechanism and optimized attention distribution effectively accelerate the model’s learning process, improving the accuracy and efficiency of super-resolution reconstruction. As shown in Figure 7, by introducing the DAFM and DEAF, OmDF-SR exhibits higher sensitivity and accuracy in handling complex textures and fine edges in endoscopic images. Figure 7a displays an original endoscopic dataset image. For a more intuitive comparison, Figure 7b enlarges a key area from Figure 7a,c, which shows the results using the Omni-SR algorithm, and Figure 7d shows the results using the OmDF-SR algorithm. By comparing these images, the advantages of the improved algorithm in detail processing and texture enhancement are evident. Particularly in the edge and highly textured regions, the OmDF-SR algorithm shows clearer boundaries and less noise, not only enhancing the visual effect of these areas but also improving the overall texture of the image, making clinical diagnosis more convenient and accurate.

To further quantify the effectiveness of the super-resolution processing, this study uses two quantitative metrics, Peak Signal-to-Noise Ratio (PSNR) and Structural Similarity Index (SSIM), to validate the performance of the OmDF-SR algorithm in the actual application of endoscopic image super-resolution, particularly in terms of detail recovery and visual quality enhancement. PSNR measures the similarity between the reconstructed image and the original image at the pixel level, with higher values indicating that the image quality is closer to the original. SSIM evaluates the similarity in structural information, luminance, and contrast of the images, which more closely aligns with human perception of image quality as shown in Table 1:

At a magnification level of ×2 and ×4, the processed image sizes increase to 1280 × 960 pixels and 2560 × 1920 pixels, respectively. At a magnification level of ×2, OmDF-SR (38.2902/0.9746 PSNR/SSIM) demonstrates superior image quality and detail recovery. As the magnification level increases, the PSNR and SSIM values for all algorithms generally decrease, due to the increased reconstruction errors and challenges at higher magnifications. Nevertheless, OmDF-SR still performs exceptionally well (32.1723/0.9489 PSNR/SSIM), outperforming Omni-SR (32.1052/0.9469 PSNR/SSIM) and other reference algorithms. This highlights the practical value and application potential of the OmDF-SR algorithm in endoscopic image super-resolution.

### 3.3. Three-Dimensional Reconstruction Experiment

This section aims to evaluate the impact of image super-resolution processing on 3D reconstruction performance. The experimental design includes two comparison groups: one group underwent OmDF-SR preprocessing, while the other did not. To ensure consistency in experimental conditions, all images were sourced from the same endoscopic image dataset. Before the experiment, all images were cropped, with brightness normalized to eliminate variations caused by different lighting conditions and image sizes. The reconstruction process employed Structure from Motion (SfM) and Multi-View Stereo (MVS) techniques, with SfM used to estimate the camera motion path and scene structure, and MVS used to reconstruct a dense 3D point cloud from multiple viewpoints.

**Figure 8:**
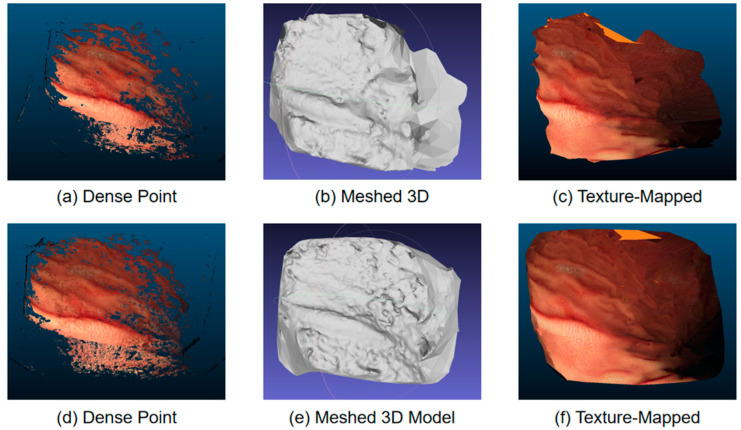
Reconstructed 3D model.

The reconstructed models are shown in Figure 8, where Figure 8a–c represent models without image super-resolution preprocessing, and Figure 8d–f represent models with preprocessing. According to qualitative evaluation, the 3D models with preprocessing exhibit superior point cloud density, meshing quality, and texture mapping richness compared to those without preprocessing. The dense point cloud model with preprocessing (Figure 8d) demonstrates higher point cloud density and lower noise levels, which are crucial for subsequent meshing and texture mapping. The meshed 3D model (Figure 8e) and the textured model (Figure 8f) both show finer structural details and higher visual realism, providing a more accurate foundation for further clinical applications and academic research.

Through comparative analysis, it is evident that preprocessing significantly enhances the accuracy and quality of reconstruction in complex scenes. This improvement is not only reflected in the visual differences but also in quantitative metrics, such as the number and density of vertices.

As shown in Table 2, the models with OmDF-SR super-resolution preprocessing exhibit higher vertex count, face count, and density compared to the datasets without preprocessing and those processed by other algorithms. This significant improvement reflects the effectiveness of OmDF-SR in enhancing image details and structural information, allowing the reconstruction process to more accurately capture the complexity of the scene. Additionally, the quality of texture mapping has also been significantly improved. The number of texture blocks in the preprocessed models has increased substantially, and in conjunction with higher resolution, this enhances the richness of surface details and visual effects. This not only improves the visual realism of the models but also enhances their practicality in medical applications such as surgical simulation and diagnostic support.

## 4. Discussion

This study has significantly enhanced the precision and efficiency of super-resolution processing and 3D reconstruction of endoscopic images through the development of the OmDF-SR, providing crucial support for clinical diagnosis and surgical planning. The OmDF-SR was trained using an endoscopic dataset, achieving a PSNR of 38.2902 dB at a magnification of ×2 and 32.1723 dB at ×4, and improving SSIM to 0.9746 at ×2 and 0.9489 at x4. Following super-resolution processing, the visual quality, point cloud density, mesh quality, and texture mapping richness of the 3D reconstructions were all significantly improved. Despite excellent performance in many aspects, the OmDF-SR also presents some issues and challenges that need further exploration.

Firstly, although this study has made significant progress in image quality and reconstruction detail, the OmDF-SR algorithm still faces challenges when dealing with extremely complex or low-quality images. High noise levels and uneven lighting conditions in the images may affect the performance of the algorithm, suggesting that further optimization is needed in future work to enhance its robustness under less-than-ideal conditions.

Secondly, although the deep learning models used in this study effectively enhance processing efficiency, they still suffer from long training times (74 h for 600 training rounds) and high computational resource demands, which limit their application in some resource-constrained clinical environments. In the future, we plan to explore more efficient model architectures and algorithm optimization strategies to reduce the dependency on computational resources, making them more suitable for rapid clinical deployment.

Moreover, although the effectiveness of this study’s results has been verified in a laboratory environment, real-world application scenarios are often more complex and variable. Therefore, future research will include extensive testing in various clinical environments to assess and validate the performance and applicability of the algorithm in actual medical operations.

Lastly, this study primarily focuses on endoscopic images, particularly in processing high-texture areas. Although OmDF-SR performs excellently in this specific application domain, its generalizability and effectiveness in processing other types of medical images still need further validation. Future work will explore applying this technology to a broader range of medical imaging types, such as MR and CT, to fully assess its wide applicability in the field of medical image processing.

Through continuous technological innovation and experimental validation, we expect the OmDF-SR algorithm to have a broader impact on the field of medical imaging, particularly in enhancing the accuracy and efficiency of image analysis and clinical decision-making, ultimately advancing the realization of precision medicine and personalized treatment strategies.

## Figures and Tables

**Table 1:** Quantitative comparison (PSNR/SSIM) with state-of-the-art methods on the same datasets.

Method	Scale	PSNR/SSIM
VDSR [27]	×2	37.3837/0.9669
CARN [28]	×2	37.7601/0.9698
PASSR [29]	×2	37.8237/0.9701
RLFN [30]	×2	38.1583/0.9722
Omni-SR [13]	×2	38.2154/0.9733
OmDF-SR	×2	38.2902/0.9746
VDSR	×4	31.1783/0.9418
CARN	×4	31.6334/0.9438
PASSR	×4	31.4625/0.9422
RLFN	×4	31.9284/0.9461
Omni-SR	×4	32.1052/0.9469
OmDF-SR	×4	32.1723/0.9489

**Table 2:** Comparison of 3D reconstruction results.

Parameter	Unprocessed	PASSR	RLFN	Omni-SR	OmDF-SR
Vertices	11,557	12,247	12,541	14,858	15,290
Faces	23,110	14,832	25,027	29,763	30,547
Texture Blocks	600	677	693	812	867
Density	368.01	429	414	483	526.23

## Data Availability

https://davidrecasens.github.io/EndoDepthAndMotion/ (accessed on 20 December 2023).
